# Assessment of Structural and Process Readiness for Postpartum Haemorrhage Care in Uganda and Ghana: A Mixed Methods Study

**DOI:** 10.1111/1471-0528.17953

**Published:** 2024-09-19

**Authors:** Tara Tancred, Andrew D. Weeks, Vincent Mubangizi, Emmanuel Nene Dei, Sylvia Natukunda, Chloe Cobb, Imelda Bates, Lucy Asamoah‐Akuoko, Bernard Natukunda

**Affiliations:** ^1^ Department of International Public Health Liverpool School of Tropical Medicine Liverpool UK; ^2^ Department of Women's and Children's Health University of Liverpool Liverpool UK; ^3^ Department of Family Medicine and Community Practice Mbarara University of Science and Technology Mbarara Uganda; ^4^ Research, Planning, Monitoring and Evaluation Department National Blood Service Ghana Accra Ghana; ^5^ Community Health Department Mbarara University of Science and Technology Mbarara Uganda; ^6^ Department of Medical Laboratory Science Mbarara University of Science and Technology Mbarara Uganda

**Keywords:** caesarean, facility readiness, Ghana, postpartum haemorrhage, transfusion, Uganda

## Abstract

**Objective:**

To determine structural and process readiness for postpartum haemorrhage (PPH) care at referral‐level facilities in Ghana and Uganda to identify opportunities for strengthening.

**Design:**

Mixed‐methods cross‐sectional study.

**Setting:**

Three districts in Ghana and two in Uganda.

**Population or Sample:**

Nine hospitals in Ghana and seven in Uganda; all hospitals had theoretical capacity for caesarean section and blood transfusion.

**Methods:**

We deployed a modular quantitative health facility assessment to explore structural readiness (drugs, equipment, staff) complemented by in‐depth interviews with maternity health service providers to understand process readiness (knowledge, attitudes, and practices as related to World Health Organization [WHO] guidance on PPH care).

**Main Outcome Measures:**

Availability of essential structural components needed to support key PPH processes of care.

**Results:**

In both countries, there was generally good structural readiness for PPH care. However, key common gaps included inadequate staffing (especially specialist physicians), and unavailability of blood for transfusion. Interviews highlighted particularly good process readiness in the provision of uterotonics, recognising and responding to retained placenta, and repairing tears. However, there were clear gaps in the utilisation of tranexamic acid and uterine balloon tamponade.

**Conclusions:**

We have identified good structural and process readiness across both Ghanaian and Ugandan health facilities to support PPH responses. However, some key missed opportunities—to align with current WHO guidance on providing bundles of interventions for PPH care—could be strengthened with minimal investment but promising impact.

## Abstract 2

**Linked article:** This article is commented on by Maclean pp. 444–445 in this issue. To view this article visit https://doi.org/10.1111/1471‐0528.17998.

## 1 Introduction

Primary postpartum haemorrhage (PPH) involves blood loss of 500 mL or more within 24 h post‐birth [1], though any blood loss accompanied by haemodynamic symptoms may qualify as PPH [2]. Global estimates indicate PPH complicates roughly 6% of births and accounts for about 20% of maternal mortality, being particularly prevalent in low‐resource settings [3, 4]. In Sub‐Saharan Africa, where maternal mortality is highest globally at 545 per 100 000 live births, PPH contributes to nearly 30% of maternal deaths [5, 6]. In both Ghana and Uganda, PPH remains the leading direct cause of maternal mortality [7, 8].

Preventing PPH is crucial [9], with an emphasis on antenatal anaemia treatment [10] and active management of the third stage of labour [11], including timely uterotonic administration [12]. In Ghana and Uganda, emphasis has been placed on the latter, though a persistently high prevalence of home births constrains effective management of the third stage of labour [13, 14]. Anaemia significantly elevates PPH risk and severity [15, 16, 17], with severely anaemic women facing higher mortality odds [18, 19]. When PPH does occur, it is essential to call for help, stabilise the patient with fluids and oxygen, and then assess and manage the cause [20, 21]. In response to blood loss, some women may require a blood transfusion or blood products as a lifesaving intervention [20].

Most PPH is preventable, manageable, and treatable. However, quality PPH care hinges on availability of structural components (e.g., infrastructure, skilled providers, drugs, equipment) and use of evidence‐based processes [22]. To explore opportunities for strengthening PPH care, we evaluated structural and process readiness in district‐level facilities across two districts in Ghana and Uganda.

## 2 Methods

### 2.1 Study Design

We carried out a mixed‐methods situational analysis of the structures and processes affecting the prevention, management, and treatment of PPH in Uganda and Ghana. The World Health Organization (WHO) has produced comprehensive guidance on steps to manage PPH (Table 1) [21], which were used to guide our data collection, analysis, and presentation of results.

**TABLE 1:** Steps in PPH care according to WHO guidelines [17].

	Step	Structural requirements	Process requirement
Initial management	Call for Help	Adequate staff to respond	Correct diagnosis of PPH Communication system
	*Resuscitation*: Administer oxygen and intravenous (IV) fluids	Oxygen tanks, masks, tubes and bags Crystalloid fluid bags IV lines and cannulas Bed capacity	Cannulation skills Correct dose
	Uterine massage	N/A	Skill
	Administer IV oxytocin	Drug availability Fridges IV lines, cannulas, needles	Cannulation skills Refrigerator checks Correct dose
	Administer IV ergometrine or sublingual misoprostol	Drug availability Fridges IV lines, cannulas, needles	Cannulation skills Refrigerator checks Correct dose
	Administer tranexamic acid	Drug availability	Correct dose
In the case of uterine atony	Intrauterine balloon tamponade	Device availability	Specialist skill
	Uterine artery embolisation	Theatre availability Surgical equipment Radiology specialist	Technical skill
	Bimanual uterine compression	Theatre availability Gynaecological gloves Specialist obstetrician	Specialist skill Referral plan
	External aortic compression	Staffing	Specialist skill Referral plan
In the case of retained placenta	IV/IM oxytocin + controlled cord traction	Drug availability Fridge	Cannulation skills Refrigerator checks Skill in controlled cord traction Correct diagnosis of cause
	Manual removal of placenta	Gynaecological gloves Theatre availability Specialist clinicians Anaesthesia	Specialist skills
	Antibiotic administration	Drug availability	Correct dose
In the case of trauma	Tear repair	Examination light Speculum Sutures and suturing needles	Skill
In the case of failure to manage	Referrals	Ambulance, driver, and fuel availability Chaperone Referral protocol	Communication between facilities
	Non‐pneumatic anti‐shock garments	Device availability	Referral plan
	Hysterectomy	Theatre availability Surgical equipment Specialist clinicians	Surgery skill Anaesthetic skill Postoperative monitoring skills

### 2.2 Study Sites

We collected data from two districts in each country. These districts were chosen to be ‘mid‐range’ in terms of population and geography to support transferability of findings. To ensure consistency, districts shared the same regional or zonal blood collection centre. All facilities classified as having capacity for surgery and blood transfusion in each district were included.

### 2.3 Data Collection

We carried out an adapted ‘service availability and readiness assessment’ [23] in study facilities. This assessment comprised modules on the availability of: standards or protocols; medicines; equipment; trained staff; and infrastructure to support PPH prevention, management, and treatment. Different modules were administered to maternal health care providers, laboratory technicians, or pharmacists as appropriate. Where needed, observation (e.g., physically viewing a medicine) took place, though to maintain clinical confidentiality, no processes of care were observed. We piloted data collection tools in an additional district in Ghana, and an additional facility in district 1 in Uganda. Our tools needed very minimal revision following piloting; therefore, data from the pilots are included in our analysis.

In‐depth interviews (IDIs) were conducted with maternity service providers, including maternity in‐charges, doctors, midwives, and the heads of the participating health facilities to understand processes of PPH care and perception of structures (staff, resources, facilities). The interview guides were developed from the WHO guidance [21] and the protocol by Akter et al. [24] All participants were purposively sampled based on their roles and their experiences relevant to PPH. IDIs took 45–60 min to complete and were carried out in English. All qualitative data were collected by skilled research assistants with at least three‐years' experience carrying out health research within the study districts.

### 2.4 Analysis

Quantitative data were analysed in Excel to generate basic descriptive statistics. Qualitative data were read and re‐read for familiarity and framework analysis was carried out [25]. Our overarching coding framework was developed from our interview guides, emphasising PPH care steps, and knowledge, attitudes, and perceptions, particularly around strengths and weaknesses relevant to each step. We triangulated data from the health facility assessments and qualitative sources to present an overall picture of structural and process readiness for PPH care across our study sites in Ghana and Uganda.

### 2.5 Ethics Statement

We received approvals from the Ghana Health Service Ethics Review Committee, and in Uganda, from the Research Ethics Committee of Mbarara University of Science and Technology and the Uganda National Council for Science and Technology. Research ethics approval was also obtained from the Liverpool School of Tropical Medicine.

Permission to carry out the research was obtained from the national, regional, and district health offices and from the head of each participating health facility. All participants provided written informed consent.

## 3 Results

### 3.1 Facility and Participant Characteristics

Data were collected from nine health facilities Ghana and seven in Uganda (Table 2), inclusive of one pilot facility in each country. Included facilities had variable capacities.

**TABLE 2:** Health facilities included in health facility assessments.

	Total facility clients per year	Number of beds
Ghana		
District 1		
Health facility 1	72 000	79
Health facility 2	156 000	126
Health facility 3	48 115	60
Health facility 4	9451	16
District 2		
Health facility 1	40 511	64
Health facility 2	30 000	100
Health facility 3	42 580	70
Health facility 4	36 000	18
District 3 (pilot district)		
Health facility 1	57 707	300
Uganda		
District 1		
Health facility 1	19 000	100
Health facility 2	500^a^	68
Health facility 3	51 100	400
Pilot facility	1200	57
District 2		
Health facility 1	30 000	100
Health facility 2	24 000	120
Health facility 3	37 200	300

^a^ A private facility with low client numbers.

Qualitative data collection from in‐depth interviews are summarised in Table 3 below. Altogether, we collected data from 23 participants in Ghana and 17 in Uganda.

**TABLE 3:** Summary of qualitative data collection.

	Number of participants
Ghana	
District 1	
In‐depth interviews: health facility 1	3
In‐depth interviews: health facility 2	3
In‐depth interviews: health facility 3	2
In‐depth interviews: health facility 4	3
Total	11
District 2	
In‐depth interviews: health facility 1	3
In‐depth interviews: health facility 2	3
In‐depth interviews: health facility 3	3
In‐depth interviews: health facility 4	3
Total	12
Total (all Ghana participants)	23
Uganda	
District 1	
In‐depth interviews: health facility 1	2
In‐depth interviews: health facility 2	3
In‐depth interviews: health facility 3	4
Total	**9**
District 2	
In‐depth interviews: health facility 1	3
In‐depth interviews: health facility 2	3
In‐depth interviews: health facility 3	2
Total	8
Total (all Uganda participants)	17

### 3.2 Identifying PPH

In the facility assessment, respondents in 7/9 (78%) of facilities in Ghana and 7/7 (100%) in Uganda described the approach to measuring blood loss after childbirth as ‘collecting blood and estimating volume’. This typically involved using a plastic sheet to collect blood and then transferring it to a measuring jar. Counting or weighing soaked cotton pads was reported in 4/9 (44%) of facilities in Ghana but none in Uganda. Visual estimation was only reported used in one facility in Ghana (Table 4).

**TABLE 4:** Availability of key structural items for PPH identification, management, and treatment.

	Ghana			Uganda		
	Available and seen	Not seen (reported available)	Not available	Available and seen	Not seen (reported available)	Not available
Blood loss estimation						
Large pads to collect blood	6/9 (67%)	1/9 (11%)	2/9 (22%)	0	0	7/7, 100%
Plastic sheets and bucket for blood collection	7/9 (78%)	2/9 (22%)	0/9	2/7 (29%)	5/7 (71%)	0
PPH care						
Urinary catheters	8/9 (89%)	1/9 (11%)	0/9	5/7 (71%)	0	2/7 (29%)
Needles	9/9 (100%)	0/9	0/9	3/7 (43%)	4/7 (57%)	0
Cannulas	9/9 (100%)	0/9	0/9	7/7 (100%)	0	0
IV infusion sets	9/9 (100%)	0/9	0/9	7/7 (100%)	0	0
IV fluids (9% sodium chloride)	9/9 (100%)	0/9	0/9	7/7 (100%)	0	0
Oxygen tanks	5/9 (56%)	4/9 (44%)	0/9	1/7 (14%)	4/7 (57%)	2/7 (29%)
Masks and tubing	6/9 (67%)	3/9 (33%)	0/9	1/7 (14%)	5/7 (71%)	1/7 (14%)
Oxytocin	9/9 (11%)	0	0	7/7 (100%)	0	0
Misoprostol	8/9 (89%)	1/9 (11%)	0/9	5/7 (71%)	1/7 (14%)	1/7 (14%)
Ergometrine	4/9 (44%)	0/9	5/9 (56%)	3/7 (43%)	1/7 (14%)	3/7 (43%)
Tranexamic acid	5/9 (56%)	3/9 (33%)	1/9 (11%)	0	5/7 (71%)	2/7 (29%)
Refrigeration (with power supply, temperature monitoring system, and alarm)	3/9 (33%)	0/9	6/9 (67%)	7/7 (100%)	0	0
Sutures and suturing needles	8/9 (89%)	1/9 (11%)	0/9	3/7 (43%)	4/7 (57%)	0
Uterine tamponade devices	5/9 (56%)	1/9 (11%)	3/9 (33%)	0	0	7/7, 100%
Protocol for management of PPH	9/9 (100%)	0/9	0/9	4/7 (57%)	3/7 (43%)	0
Referral protocol for PPH	9/9 (100%)	0/9	0/9	0	3/7 (43%)	4/7 (57%)
Specialist care availability	Always available	Sometimes available	Never available	Always available	Sometimes available	Never available
Obstetric specialist	2/9 (22%)	1/9 (11%)	6/9 (67%)	3/7 (43%)	0	4/7 (57%)
Anaesthetist	0/9	0/9	9/9 (100%)	3/7 (43%)	2/7 (29%)	2/7 (29%)
Functional operating theatre	9/9 (100%)	0/9	0/9	7/7 (100%)	0	0

All facilities in Ghana had adequate supplies for at least one method of objective measurement of blood loss. In all facilities in Uganda, plastic sheets and collecting dishes were either seen or reported as available, but large pads for collecting blood were not available (Table 4).

In interviews, almost all maternity service providers in Ghana and most in Uganda mentioned counting soaked pads to estimate blood loss. However, in Uganda, large pads were not available in any facilities according to health facility assessments, contrasting with the reported practice. Using a measuring jar/cup was a common response in both countries. Just under half of participants in both countries indicated reliance on visual estimation—again contrasting with health facility assessments—with some in Ghana noting that experienced clinicians might use this method more often.

We know the weight of the bed mat, the unsoaked ones, so when the bed mat is soaked what we do is…we carry the bed mat, put it on a scale and then weigh it so depending on the kilograms or grams we get then we equate it to millilitres [of blood] (IDI, maternity in‐charge, Ghana).

Interviewer So how is [blood loss] estimated?

Respondent We just see if it is a lot (IDI, midwife, Uganda).

In Uganda, most maternity service providers made the distinction between PPH volumes of blood loss for vaginal births versus caesarean sections—500 mL considered PPH in the former and 1000 mL in the latter. In Ghana, however, in terms of a cut‐off for PPH, participants commonly mentioned a range from 250 to 400 mL of blood loss with a reliance on accompanying clinical symptoms, with only one participant suggesting it was 500 mL.

It depends on the health of the patient. At times somebody will bleed from even 300, 400, 500 ml but looking at the person and how she is, if the patient is not all that pale and she bleeds about 300, nothing will happen, but if the patient is pale and not healthy and bleeds up to 300, 400 it is taken as PPH. But if the patient is healthy and she bleeds up to 500, no matter what, she's termed as having PPH and we try to manage (IDI, maternity in‐charge, Ghana).

### 3.3 PPH Management

#### 3.3.1 Initial Management

##### 3.3.1.1 Call for Help

When responding to a PPH emergency, most respondents in both countries indicated that they would first ‘shout for help’.

First step, you call for help, shout for help…even if it's a cleaner, please come (IDI, doctor, Uganda).

##### 3.3.1.2 Resuscitation Through Oxygen and IV Fluids

Intravenous fluids (0.9% sodium chloride) were available across all facilities in both countries in our assessment. Oxygen tanks, tubing and masks were reported available (or were ‘available and seen’) in 9/9 (100%) of facilities in Ghana, but only 5/7 (71%) in Uganda (Table 4). Maternity service providers in both countries emphasised the importance of early introduction of fluid resuscitation following blood loss. However, doing vital observation and giving oxygen was rarely mentioned.

Most participants also noted the importance of finding the cause of the PPH and choosing a course of action on that basis.

…immediately we get such a case [of PPH] after we've estimated the blood loss, quickly we set up 2 IV lines, we hydrate the client with 2 litres of normal saline, before then we take samples for [full blood count], grouping and cross matching because probably we may need a blood. Then we look for the cause of the PPH (IDI, maternity in‐charge, Ghana).

##### 3.3.1.3 Uterine Massage

Maternity service providers regularly mentioned the importance of bladder catheterisation, stating that a full bladder can interfere with uterine contraction. Both uterine massage—which was mentioned by around half of participants in Uganda and a third of participants in Ghana—and catheterisation were described as supportive mechanisms alongside uterotonics.

After rehydrating the mother… they also give the oxytocin and they massage the uterus and they leave the bladder empty (IDI, midwife, Uganda).

Catheters were available in 5/7 (71%) of facilities sampled in Uganda and 9/9 (100%) of facilities in Ghana.

##### 3.3.1.4 Administer Uterotonics

Oxytocin was available and seen across all facilities in both countries. Misoprostol was seen or reported available in 9/9 (100%) of facilities in Ghana and 6/7 (86%) of facilities in Uganda. To store oxytocin appropriately, fully functional refrigeration (with power supply, temperature monitoring, and an alarm system) was available in all facilities in Uganda but only 3/9 facilities in Ghana. Intravenous infusion kits, cannulas and needles were available in all facilities in both countries to support uterotonic administration (Table 4).

Participants expressed confidence in their use of uterotonics, either as part of the active management of the third stage of labour, or upon onset of PPH. Oxytocin was mentioned by almost all participants in both countries, with a few also noting the use of misoprostol. Unavailability and stockouts of uterotonics were challenges mentioned by participants in both countries, which sometimes resulted in mothers having to supply their own.

The challenges that we mostly face is lack of supplies or sundries; they are not sustainable. For example, we find we don't have misoprostol and yet it is needed, then you have to tell the mother to go and buy the misoprostol (IDI, midwife, Uganda).

##### 3.3.1.5 Administer Tranexamic Acid

No participants in Uganda and only three in Ghana described the use of tranexamic acid in PPH care, despite it being reported available in most facilities (5/7, 71% in Uganda and 8/9, 81% in Ghana; Table 4).

We concentrate on things to help the uterus to contract by giving uterotonics like Cytotec [misoprostol] and probably tranexamic acid in extreme bleeding cases (IDI, midwife, Ghana).

#### 3.3.2 Refractory (Persistent) Uterine Atony

Uterine balloon tamponade was the only approach specific to uterine atony investigated in our assessment. Necessary equipment was available (available and seen or reported available) in 6/9 (67%) facilities in Ghana and no facilities in Uganda (Table 4).

The use of uterine balloon tamponade was sparingly mentioned by participants. Only one participant in Uganda mentioned it, specifically noting that it was *not* used, and only one participant in Ghana said it was used.

Of recent, we have also learnt of balloon tamponade but it is…unfortunate that we have not tried it out, so I can't comment on it (IDI, doctor, Uganda).

Bimanual compression, external aortic compression, and uterine artery embolisation were not mentioned by any participants in either country.

#### 3.3.3 Retained Placenta

Management of retained placenta, including manual removal if necessary, was cited across almost all maternity service providers in both countries. However, in both contexts, gynaecological gloves for manual removal were frequently unavailable. No participants mentioned the administration of antibiotics to support infection prevention.

Sometimes you find we are lacking gynaecological gloves, the long ones we use to reach the uterus (IDI, midwife, Uganda).

#### 3.3.4 Management of Trauma

Finding and suturing (predominantly cervical) tears was also mentioned by most maternity service providers in both countries and suturing equipment was reported available in all facilities to manage tears (Table 4).

…Trauma is about the ruptured uterus. It may [also] be any cervical tear of any degree, or anything related to tearing (IDI, midwife, Uganda).

Assessing and repairing the uterus specifically was only mentioned by around a third of participants in Uganda and sparingly by participants in Ghana, mostly concentrated amongst the physicians who might be more likely to use these techniques.

#### 3.3.5 Hysterectomy

The use of hysterectomy was indicated by only two maternity service providers in Uganda, both suggesting it should be used ‘if all else fails’. Only one participant in Ghana mentioned use of hysterectomy.

If we do all these things and the bleeding is still coming then what we have to do is to take out the uterus…Because we say that the life of the woman is better to keep than to keep the uterus for the woman to die, so we sacrifice the uterus (IDI, doctor, Ghana).

To carry out manual removal of placenta, uterine repair, or hysterectomy, a functional operating theatre was ‘always available’ across all facilities in both countries (Table 4).

#### 3.3.6 Blood Transfusion

Roughly, half of the maternity service providers in both countries reported sending a blood sample for haemoglobin estimation and for blood grouping and cross‐matching in case a blood transfusion was needed. In both contexts, participants were very clear that ≥1000 mL of blood loss would typically necessitate a blood transfusion. Lack of blood for transfusion, particularly rhesus negative blood, was mentioned as one of the biggest challenges to PPH care by almost all participants in both countries.

You find most of the time we don't have blood telling the truth…blood is not readily available that is a barrier number one that I can mention, I think there is no other barrier (IDI, facility in‐charge, Uganda).

#### 3.3.7 Referral

Referrals were widely mentioned amongst maternity service providers as necessary when it was not possible to manage a PPH at the facility given the current resources or staff available. Across both countries, overwhelmingly, referrals were cited as needed when blood was not available. To a much lesser extent, lack of availability of specialist clinicians or a functional operating theatre were also cited as prominent reasons for referral.

If we don't have the blood available then we don't have any option than to refer to a higher facility (IDI, maternity in‐charge, Ghana).

### 3.4 Other Structural Elements

#### 3.4.1 Availability of Standards and Protocols

PPH protocols were available in all study sites, and clear guidelines were integrated into posters on the walls of delivery suites. This also included facility phone numbers to support referrals.

#### 3.4.2 Staffing and Team‐Working

Understaffing, notably a lack of specialists, was consistently cited by maternity service providers in both countries and confirmed by health facility assessments. Obstetric specialists were absent in 6/9 (67%) of facilities in Ghana and 4/7 (57%) in Uganda. Non‐specialist staff, typically medical officers, assumed their roles, such as performing caesarean sections and overseeing PPH management and blood transfusions. Specialist anaesthetists were absent in all facilities in Ghana and only consistently present in 3/7 (43%) of facilities in Uganda, with nurse anaesthetists in Ghana and anaesthetist officers in Uganda filling this role instead.

The main barrier…is understaffing. If there are limited staff and they are overwhelmed by the number of mothers delivering here and there, someone out there may be seriously bleeding and is not being attended to (IDI, doctor, Uganda).

Several participants, especially in Uganda, recognised that a lack of obstetric specialists placed a greater responsibility on midwives. The more senior specialists were concerned that midwives may not have appropriate skills or competencies, which could lead to incorrect execution of PPH management guidelines.

If the doctor is around, he is the one that does it, if he is not around, then you have to do it (IDI, midwife, Uganda).

## 4 Discussion

### 4.1 Main Findings

This study revealed generally good structural readiness for PPH care in both countries. However, common gaps were identified, including poor availability of gynaecological gloves, inadequate staffing (especially specialist physicians), and unavailability of blood. Supplies for uterine balloon tamponade were unavailable in Uganda. Interviews with maternity care providers highlighted particularly good process readiness in the provision of uterotonics, recognising and responding to retained placenta, and repairing tears. However, in both contexts, there were clear gaps in the utilisation of tranexamic acid and uterine balloon tamponade.

### 4.2 Interpretation

Accurate evaluation and documentation of blood loss is crucial for timely intervention [26, 27, 28]. Recent studies suggest the use of calibrated drapes for objective measurement [29], as visual estimation is often inaccurate and delays response [26, 30, 31, 32, 33]. However, objective measures may sometimes overestimate blood loss due to fluid presence or underestimate it if blood is not captured effectively. Interpretation of objective results relative to the clinical context is critical; the significance of a small amount of blood loss in already‐anaemic women should not be overlooked. Therefore, alongside volume measurement, assessing clinical indicators of hypovolaemia and severe anaemia is essential and may facilitate timely intervention [26, 27, 31, 34], as observed by some participants, particularly in Ghana.

At least one uterotonic was available in all facilities, though oxytocin is not heat stable and requires refrigeration. Adequate refrigeration was not present across all facilities in Ghana. Alternatives like misoprostol and carbetocin—a heat‐stable, affordable, long‐acting uterotonic found to be as effective as oxytocin for PPH prevention—may be useful [35, 36, 37, 38, 39].

Tranexamic acid reduces the risk of mortality due to PPH by roughly 30% when administered within three hours of the onset of PPH and reduces the need for further interventions [40, 41, 42, 43, 44]. Tranexamic acid was available across most facilities, but rarely (if ever) mentioned for its role in PPH management. Its underuse has been reported in other low‐resource settings [45]. Staff sensitisation around use of tranexamic acid may, therefore, be a feasible, low‐cost, and effective area of intervention to improve PPH outcomes. Further to this, emerging research suggests that *early* provision of tranexamic acid to women who are at the highest risk of PPH (e.g., those with clinically significant anaemia) as a quasi‐prophylactic measure may be of value, including during caesarean section [46, 47].

There was also very little use of uterine balloon tamponade. The WHO recommends its use where uterine atony has been determined as the cause of PPH where first‐line treatments for PPH have been provided and the patient can be adequately monitored [48]. Though some evidence has been poor quality or inconclusive [49], some studies have found it to be effective for haemorrhage cessation [50, 51, 52]. Improvised and low‐cost devices (e.g., using condom‐catheter tamponade [53]) are now widely available, which may be yet another under‐utilised intervention that could support PPH care in both countries [9, 54].

The bottlenecks we identified in our study are echoed in low‐resource settings globally [24]. Most notably, the lack of specialist physicians hampers advanced PPH care—such as hysterectomy—and may result in treatment delays while the patient is transferred to another facility [24, 55]. To tackle these bottlenecks, the WHO emphasises supporting PPH care through national policy, improved procurement, and training to address staff shortages—actions that would be beneficial across our study settings [56].

Since our study, the WHO has released new PPH guidelines advocating for routine blood loss measurement for *all* vaginal births. For detected PPH, a first‐line care bundle is recommended, including use of uterine massage, oxytocic agent and tranexamic acid administration, IV fluids, genital tract examination, and escalation of care within 15 min [1]. If bleeding persists, escalation to senior clinicians or referral is advised. The ‘response to refractory PPH bundle’ for persistent PPH then includes uterine compression, anti‐shock garment use, and intrauterine balloon tamponade insertion [57]. Standardised implementation may be challenging, so clear protocols, training, and resource availability will be essential. Our findings indicate facilities are likely equipped for the first‐line bundle, but tranexamic acid usage may need more staff awareness and training. The ‘response to refractory PPH’ bundle seems likely to be underused, as its interventions were rarely or never discussed by participants, suggesting limited knowledge and/or use.

### 4.3 Strengths and Limitations

This mixed‐methods study, spanning multiple districts in two countries, involved 16 referral‐level health facilities. We triangulated both qualitative data and findings from a thorough health facility assessment. However, we acknowledge some key limitations: We did not explicitly inquire about each step in PPH care according to WHO guidelines. Instead, we aimed for participants to reflect on their most common practices, potentially indicating areas for improvement. Consequently, some steps, such as uterine artery embolisation or use of hypovolemic shock garments, though not mentioned, were not explicitly asked about. While our results suggest these practices may not be common, they could simply be underreported. Further, our scope was intentionally broad to deliver a snapshot of PPH practices, though this necessarily constrained an in‐depth exploration of each step. Finally, we recognise that participants in qualitative research might have a higher level of knowledge or interest in PPH, potentially skewing results.

## 5 Conclusion

We have identified good structural and process readiness across both Ghanaian and Ugandan health facilities to support PPH responses. However, some key bottlenecks and missed opportunities were commonly reflected in both contexts that could be strengthened with minimal investment whilst contributing to significant gains. These include supporting staff to assess both the volume and severity of blood loss for timely PPH intervention and strengthening use of tranexamic acid and uterine balloon tamponade. Higher‐level challenges around staffing and blood availability may require greater investment to resolve. Future research should explore readiness around each component of the first‐line and refractory care bundles for PPH now recommended by the WHO.

## Author Contributions

T.T., A.D.W., I.B., L.A.‐A., and B.N. conceptualised the study, T.T., A.D.W., V.M., E.N.D., S.N., I.B., L.A.‐A., and B.N. drafted data collection instruments and supported preliminary analysis. T.T. and C.C. led the detailed analysis. T.T. wrote the first draft of the manuscript. All authors contributed to writing of subsequent drafts.

## Conflicts of Interest

The authors declare no conflicts of interest.

## Data Availability

Anonymised data from this study can be made available on reasonable request to the corresponding author.

## References

[1] World Health Organization , WHO Recommendations on the Assessment of Postpartum Blood Loss and Use of a Treatment Bundle for Postpartum Haemorrhage (Geneva, Switzerland: World Health Organization, Human Reproduction Programme, 2023). 38170808

[2] E. Mavrides , S. Allard , E. Chandraharan , et al., “Prevention and Management of Postpartum Haemorrhage,” BJOG: An International Journal of Obstetrics and Gynaecology 124 (2016): e106–e149. 27981719 10.1111/1471-0528.14178

[3] G. Carroli , C. Cuesta , E. Abalos , and A. M. Gulmezoglu , “Epidemiology of Postpartum Haemorrhage: A Systematic Review,” Best Practice and Research Clinical Obstetrics and Gynaecology 22, no. 6 (2008): 999–1012. 18819848 10.1016/j.bpobgyn.2008.08.004

[4] L. Say , D. Chou , A. Gemmill , et al., “Global Causes of Maternal Death: A WHO Systematic Analysis,” Lancet Global Health 2, no. 6 (2014): e323–e333. 25103301 10.1016/S2214-109X(14)70227-X

[5] World Health Organization, UNICEF, UNFPA, World Bank Group , UNDESA Population Division. Trends in Maternal Mortality 2000 to 2020: Estimates by WHO, UNICEF, UNFPA, World Bank Group and UNDESA/Population Division (Geneva, Switzerland: World Health Organization, 2023). Report No.: 9240068759.

[6] R. Musarandega , M. Nyakura , R. Machekano , R. Pattinson , and S. P. Munjanja , “Causes of Maternal Mortality in Sub‐Saharan Africa: A Systematic Review of Studies Published From 2015 to 2020,” Journal of Global Health 11 (2021): 04048. 34737857 10.7189/jogh.11.04048 PMC8542378

[7] J. Adu and M. F. Owusu , “How Do We Improve Maternal and Child Health Outcomes in Ghana?” International Journal of Health Planning and Management 38, no. 4 (2023): 898–903. 36974514 10.1002/hpm.3639

[8] C. Birabwa , A. Banke‐Thomas , P. Waiswa , et al., “Maternal Health in Cities: Analysis of Institutional Maternal Mortality and Health System Bottlenecks in Kampala City Uganda, 2016–2021,” Journal of Global Health Reports 8 (2024): 1–14.

[9] A. D. Weeks , “Reducing Maternal Deaths From Haemorrhage: Seeking the Low‐Hanging Fruit,” BJOG: An International Journal of Obstetrics and Gynaecology 131 (2024): 1025–1028. 38177088 10.1111/1471-0528.17753

[10] A. Butwick and N. McDonnell , “Antepartum and Postpartum Anemia: A Narrative Review,” International Journal of Obstetric Anesthesia 47 (2021): 102985. 33893005 10.1016/j.ijoa.2021.102985

[11] A. E. Ubom , O. A. Ijarotimi , S. Nyeche , P. C. Oriji , E. O. Ayegbusi , and A. Appiah‐Kubi , “A Review of Nonpharmacologic Prevention and Management of Postpartum Haemorrhage in Low‐Resource Settings,” Nigerian Journal of Medicine 32, no. 2 (2023): 109–112.

[12] G. J. Hofmeyr , N. T. Mshweshwe , and A. M. Gülmezoglu , “Controlled Cord Traction for the Third Stage of Labour,” Cochrane Database of Systematic Reviews 1 (2015): CD008020. 25631379 10.1002/14651858.CD008020.pub2 PMC6464177

[13] B. K. Opoku , “Primary Postpartum Haemorrhage: A Review of Current Treatment and Prevention Practices in Ghana,” Research Chronicle in Health Sciences 1, no. 2 (2015): 99–109.

[14] S. Ononge , F. Mirembe , J. Wandabwa , and O. M. Campbell , “Incidence and Risk Factors for Postpartum Hemorrhage in Uganda,” Reproductive Health 13 (2016): 38. 27080710 10.1186/s12978-016-0154-8 PMC4832492

[15] T. T. Lao , L. L. Wong , S. Y. A. Hui , and D. S. Sahota , “Iron Deficiency Anaemia and Atonic Postpartum Haemorrhage Following Labour,” Reproductive Sciences 1‐9 (2022): 1102–1110. 10.1007/s43032-021-00534-1 34993930

[16] M. O. Omotayo , A. I. Abioye , M. Kuyebi , and A. C. Eke , “Prenatal Anemia and Postpartum Hemorrhage Risk: A Systematic Review and Meta‐Analysis,” Journal of Obstetrics and Gynaecology Research 47, no. 8 (2021): 2565–2576. 34002432 10.1111/jog.14834 PMC9258034

[17] M. Nair , S. Chhabra , S. S. Choudhury , et al., “Relationship Between Anaemia, Coagulation Parameters During Pregnancy and Postpartum Haemorrhage at Childbirth: A Prospective Cohort Study,” BMJ Open 11, no. 10 (2021): e050815. 10.1136/bmjopen-2021-050815 PMC8491293 34607867

[18] R. Mansukhani , H. Shakur‐Still , R. Chaudhri , et al., “Maternal Anaemia and the Risk of Postpartum Haemorrhage: A Cohort Analysis of Data From the WOMAN‐2 Trial,” Lancet Global Health 11, no. 8 (2023): e1249–e1259. 37390833 10.1016/S2214-109X(23)00245-0 PMC10353972

[19] M. F. Young , “Maternal Anaemia and Risk of Mortality: A Call for Action,” Lancet Global Health 6, no. 5 (2018): e479–e480. 29571593 10.1016/S2214-109X(18)30185-2

[20] R. Collis and E. Guasch , “Managing Major Obstetric Haemorrhage: Pharmacotherapy and Transfusion,” Best Practice and Research Clinical Anaesthesiology 31, no. 1 (2017): 107–124. 28625299 10.1016/j.bpa.2017.02.001

[21] World Health Organization , WHO Recommendations for the Prevention and Treatment of Postpartum Haemorrhage (Geneva, Switzerland: World Health Organization, 2012). 23586122

[22] A. Donabedian , “Evaluating the Quality of Medical Care,” Milbank Quarterly 83, no. 4 (2005): 691–729. 16279964 10.1111/j.1468-0009.2005.00397.x PMC2690293

[23] World Health Organization , Service Availability and Readiness Assessment (SARA): An Annual Monitoring System for Service Delivery: Implementation Guide (Geneva, Switzerland: World Health Organization, 2013).

[24] S. Akter , F. Lorencatto , G. Forbes , et al., “Perceptions and Experiences of the Prevention, Identification and Management of Postpartum Haemorrhage: A Qualitative Evidence Synthesis,” Cochrane Database of Systematic Reviews 2020, no. 12 (2020): CD013795. 10.1002/14651858.CD013795.pub2 PMC10680124 38009552

[25] N. K. Gale , G. Heath , E. Cameron , S. Rashid , and S. Redwood , “Using the Framework Method for the Analysis of Qualitative Data in Multi‐Disciplinary Health Research,” BMC Medical Research Methodology 13, no. 1 (2013): 117. 24047204 10.1186/1471-2288-13-117 PMC3848812

[26] A. Hancock , A. D. Weeks , and L. D. Tina , “Assessing Blood Loss in Clinical Practice,” Best Practice and Research Clinical Obstetrics and Gynaecology 61 (2019): 28–40. 31235396 10.1016/j.bpobgyn.2019.04.004

[27] D. Katz and M. Farber , “Can Measuring Blood Loss at Delivery Reduce Hemorrhage‐Related Morbidity?” International Journal of Obstetric Anesthesia 46 (2021): 102968. 33774489 10.1016/j.ijoa.2021.102968

[28] A. Briley , P. T. Seed , G. Tydeman , et al., “Reporting Errors, Incidence and Risk Factors for Postpartum Haemorrhage and Progression to Severe PPH: A Prospective Observational Study,” BJOG: An International Journal of Obstetrics and Gynaecology 121, no. 7 (2014): 876–888. 24517180 10.1111/1471-0528.12588 PMC4282054

[29] I. Gallos , A. Devall , J. Martin , et al., “Randomized Trial of Early Detection and Treatment of Postpartum Hemorrhage,” New England Journal of Medicine 389, no. 1 (2023): 11–21. 37158447 10.1056/NEJMoa2303966

[30] L. D. Rothermel and J. M. Lipman , “Estimation of Blood Loss Is Inaccurate and Unreliable,” Surgery 160, no. 4 (2016): 946–953. 27544540 10.1016/j.surg.2016.06.006

[31] S. Meher , “How Should We Diagnose and Assess the Severity of PPH in Clinical Trials?” Best Practice and Research Clinical Obstetrics and Gynaecology 61 (2019): 41–54. 31155462 10.1016/j.bpobgyn.2019.04.003

[32] J. Lemée , A. Scalabre , C. Chauleur , and T. Raia‐Barjat , “Visual Estimation of Postpartum Blood Loss During a Simulation Training: A Prospective Study,” Journal of Gynecology Obstetrics and Human Reproduction 49, no. 4 (2020): 101673. 31816433 10.1016/j.jogoh.2019.101673

[33] S. Akter , G. Forbes , S. Miller , et al., “Detection and Management of Postpartum Haemorrhage: Qualitative Evidence on Healthcare Providers' Knowledge and Practices in Kenya, Nigeria, and South Africa,” Frontiers in Global Women's Health 3 (2022): 1020163. 10.3389/fgwh.2022.1020163 PMC9715762 36467287

[34] A. Borovac‐Pinheiro , R. Pacagnella , J. Cecatti , et al., “Postpartum Hemorrhage: New Insights for Definition and Diagnosis,” American Journal of Obstetrics and Gynecology 219, no. 2 (2018): 162–168. 29660298 10.1016/j.ajog.2018.04.013

[35] M. Widmer , G. Piaggio , T. M. Nguyen , et al., “Heat‐Stable Carbetocin Versus Oxytocin to Prevent Hemorrhage After Vaginal Birth,” New England Journal of Medicine 379, no. 8 (2018): 743–752. 29949473 10.1056/NEJMoa1805489

[36] S. S. Vernekar , S. S. Goudar , M. Metgud , et al., “Effect of Heat Stable Carbetocin vs Oxytocin for Preventing Postpartum Haemorrhage on Post Delivery Hemoglobin–a Randomized Controlled Trial,” Journal of Maternal‐Fetal and Neonatal Medicine 35, no. 25 (2022): 8744–8751. 34763599 10.1080/14767058.2021.2001799

[37] S. B. Chikkamath , G. M. Katageri , A. A. Mallapur , et al., “Duration of Third Stage Labour and Postpartum Blood Loss: A Secondary Analysis of the WHO CHAMPION Trial Data,” Reproductive Health 18, no. 1 (2021): 230. 34775959 10.1186/s12978-021-01284-8 PMC8591926

[38] R. Ghosh , O. Owa , N. Santos , et al., “Heat Stable Carbetocin or Oxytocin for Prevention of Postpartum Hemorrhage Among Women at Risk: A Secondary Analysis of the CHAMPION Trial,” International Journal of Gynecology and Obstetrics 164, no. 1 (2024): 124–130. 37357606 10.1002/ijgo.14938

[39] Ferring Pharmaceuticals , “Swissmedic approves Carbetocin Ferring for the prevention of postpartum haemorrhage in all births 2020,” https://www.ferring.com/swissmedic‐approves‐carbetocin‐ferring‐for‐the‐prevention‐of‐postpartum‐haemorrhage‐in‐all‐births/.

[40] A. Brenner , K. Ker , H. Shakur‐Still , and I. Roberts , “Tranexamic Acid for Post‐Partum Haemorrhage: What, Who and When,” Best Practice and Research Clinical Obstetrics and Gynaecology 61 (2019): 66–74. 31128974 10.1016/j.bpobgyn.2019.04.005 PMC6891248

[41] F. A. Ferrari , S. Garzon , R. Raffaelli , et al., “Tranexamic Acid for the Prevention and the Treatment of Primary Postpartum Haemorrhage: A Systematic Review,” Journal of Obstetrics and Gynaecology 42, no. 5 (2022): 734–746. 34996342 10.1080/01443615.2021.2013784

[42] H. Shakur , I. Roberts , B. Fawole , et al., “Effect of Early Tranexamic Acid Administration on Mortality, Hysterectomy, and Other Morbidities in Women With Post‐Partum Haemorrhage (WOMAN): An International, Randomised, Double‐Blind, Placebo‐Controlled Trial,” Lancet 389, no. 10084 (2017): 2105–2116. 28456509 10.1016/S0140-6736(17)30638-4 PMC5446563

[43] M. Hemani , K. Parihar , N. Gervais , and M. Morais , “Tranexamic Acid Use in the Postpartum Period Since the WOMAN Trial: A Retrospective Chart Review,” Journal of Obstetrics and Gynaecology Canada 44, no. 3 (2022): 279–285.e2. 34742944 10.1016/j.jogc.2021.10.014

[44] M. Franchini , D. Focosi , M. Zaffanello , and P. M. Mannucci , “Efficacy and Safety of Tranexamic Acid in Acute Haemorrhage,” British Medical Journal 384 (2024): e075720. 38176733 10.1136/bmj-2023-075720

[45] J. Ng'ang'a , T. Chitimbe , R. Mburu , et al., “Challenges in Updating National Guidelines and Essential Medicines Lists in sub‐Saharan African Countries to Include WHO‐Recommended Postpartum Hemorrhage Medicines,” International Journal of Gynecology and Obstetrics 158 (2022): 11–13. 35762803 10.1002/ijgo.14269 PMC9543462

[46] G. Saccone , L. Della Corte , P. D'Alessandro , et al., “Prophylactic Use of Tranexamic Acid After Vaginal Delivery Reduces the Risk of Primary Postpartum Hemorrhage,” Journal of Maternal‐Fetal and Neonatal Medicine 33, no. 19 (2020): 3368–3376. 30704334 10.1080/14767058.2019.1571576

[47] S. H. Lee , M. E. J. Kwek , S. Tagore , et al., “Tranexamic Acid, as an Adjunct to Oxytocin Prophylaxis, in the Prevention of Postpartum Haemorrhage in Women Undergoing Elective Caesarean Section: A Single‐Centre Double‐Blind Randomised Controlled Trial,” BJOG: An International Journal of Obstetrics and Gynaecology 130, no. 9 (2023): 1007–1015. 36852501 10.1111/1471-0528.17445

[48] World Health Organization , WHO recommendation on uterine balloon tamponade for the treatment of postpartum haemorrhage (Geneva, Switzerland: World Health Organization, 2021). 33909382

[49] V. Pingray , M. Widmer , A. Ciapponi , et al., “Effectiveness of Uterine Tamponade Devices for Refractory Postpartum Haemorrhage After Vaginal Birth: A Systematic Review,” BJOG: An International Journal of Obstetrics and Gynaecology 128, no. 11 (2021): 1732–1743. 34165867 10.1111/1471-0528.16819 PMC9292664

[50] M. M. Dalmedico , F. M. Barbosa , C. M. Toledo , W. A. Martins , A. R. Fedalto , and S. O. Ioshii , “Intrauterine Balloon Tamponade for Postpartum Hemorrhage,” Fisioterapia em Movimento 35 (2022): e35617.

[51] F. J. Ruiz Labarta , M. P. Pintado Recarte , L. Joigneau Prieto , et al., “Factors Associated With Failure of Bakri Balloon Tamponade for the Management of Postpartum Haemorrhage. Case Series Study and Systematic Review,” Healthcare 35 (2021): 295. 10.3390/healthcare9030295 PMC7999507 33800388

[52] M. Revert , J. Cottenet , P. Raynal , E. Cibot , C. Quantin , and P. Rozenberg , “Intrauterine Balloon Tamponade for Management of Severe Postpartum Haemorrhage in a Perinatal Network: A Prospective Cohort Study,” BJOG: An International Journal of Obstetrics and Gynaecology 124, no. 8 (2017): 1255–1262. 27781401 10.1111/1471-0528.14382

[53] A. Aderoba , B. Olagbuji , A. Akintan , O. Oyeneyin , O. Owa , and J. Osaikhuwuomwan , “Condom‐Catheter Tamponade for the Treatment of Postpartum Haemorrhage and Factors Associated With Success: A Prospective Observational Study,” BJOG: An International Journal of Obstetrics and Gynaecology 124, no. 11 (2017): 1764–1771. 27726298 10.1111/1471-0528.14361

[54] G. Hofmeyr , “Novel Concepts and Improvisation for Treating Postpartum Haemorrhage: A Narrative Review of Emerging Techniques,” Reproductive Health 20, no. 1 (2023): 116. 37568196 10.1186/s12978-023-01657-1 PMC10422815

[55] M. Owen , A. Cassidy , and A. Weeks , “Why Are Women Still Dying From Obstetric Hemorrhage? A Narrative Review of Perspectives From High and Low Resource Settings,” International Journal of Obstetric Anesthesia 46 (2021): 102982. 33903002 10.1016/j.ijoa.2021.102982

[56] World Health Organization , A Roadmap to Combat Postpartum Haemorrhage Between 2023 and 2030 (Geneva, Switzerland: World Health Organization, 2023).

[57] F. Althabe , M. N. Therrien , V. Pingray , et al., “Postpartum Hemorrhage Care Bundles to Improve Adherence to Guidelines: A WHO Technical Consultation,” International Journal of Gynecology and Obstetrics 148, no. 3 (2020): 290–299. 31709527 10.1002/ijgo.13028 PMC7064978

